# Tyrannosaurid-like osteophagy by a Triassic archosaur

**DOI:** 10.1038/s41598-018-37540-4

**Published:** 2019-01-30

**Authors:** Martin Qvarnström, Per E. Ahlberg, Grzegorz Niedźwiedzki

**Affiliations:** Department of Organismal Biology, Evolutionary Biology Centre, Uppsala University, Norbyvägen 18A, 752 36 Uppsala, Sweden

## Abstract

Here we present evidence for osteophagy in the Late Triassic archosaur *Smok wawelski* Niedźwiedzki, Sulej and Dzik, 2012, a large theropod-like predator from Poland. Ten medium to large-sized coprolites are matched, by their dimensions and by association with body fossils and footprints, to *S. wawelski*. The coprolites contain fragments of large serrated teeth as well as up to 50 percent by volume of bone fragments, with distinct fragmentation and angularity, from several prey taxa. This suggests pronounced osteophagy. Further evidence for bone-crushing behaviour is provided by isolated worn teeth, bone-rich regurgitalites (fossil regurgitates) and numerous examples of crushed or bite-marked dicynodont bones, all collected from the same bone-bearing beds in the Lipie Śląskie clay-pit. Several of the anatomical characters related to osteophagy, such as a massive head and robust body, seem to be shared by *S. wawelski* and the tyrannosaurids, despite their wide phylogenetic separation. These large predators thus provide evidence of convergence driven by similar feeding ecology at the beginning and end of the age of dinosaurs.

## Introduction

Osteophagous feeding behaviour appears to have been rare among theropod dinosaurs, judging by the preponderance of lightly built skulls and delicate blade-like teeth among them, as well as the relative rarity of tooth marks on bones in dinosaur-dominated faunas^1^. The major exception is the Late Cretaceous tyrannosaurids, which have massive skulls and robust but frequently worn teeth, and are associated with heavily bite-marked bones and bone-rich coprolites^2–5^.

A number of body plans, long considered to be restricted to later Mesozoic dinosaurs, have recently been identified in unrelated archosaur lineages of the Mid-Late Triassic. These include large predatory rauisuchians with high and narrow, or massive and posteriorly broad skulls, similar to those of large neotheropods and tyrannosaurids^6^, bipedal and toothless pseudosuchians closely resembling ornithomimosaurids^7^, dome-skulled forms similar to pachycephalosaurs^8^, and even forms which possessed a pair of anterodorsally projecting and sub-conical horns, closely resembling those of some ceratopsids^9^. This rapid early occupation of ecomorphospace, at a time when the dinosaurs proper were undergoing their earliest radiation and were not yet very diverse, bears testament to the complexity of the ecosystem but also creates problems for determining the phylogenetic affinities of some taxa. A good example is *S. wawelski* from the late Norian-earliest Rhaetian of Poland^10,11^. At an estimated total length of 5–6 meters, this is the largest predatory archosaur known from the Late Triassic of Europe. It was apparently bipedal and has a somewhat theropod-like overall *gestalt*, but its anatomy combines dinosaur-like, rauisuchian-like and primitive archosaur characters in an incongruous manner^11^.

**Figure 1:**
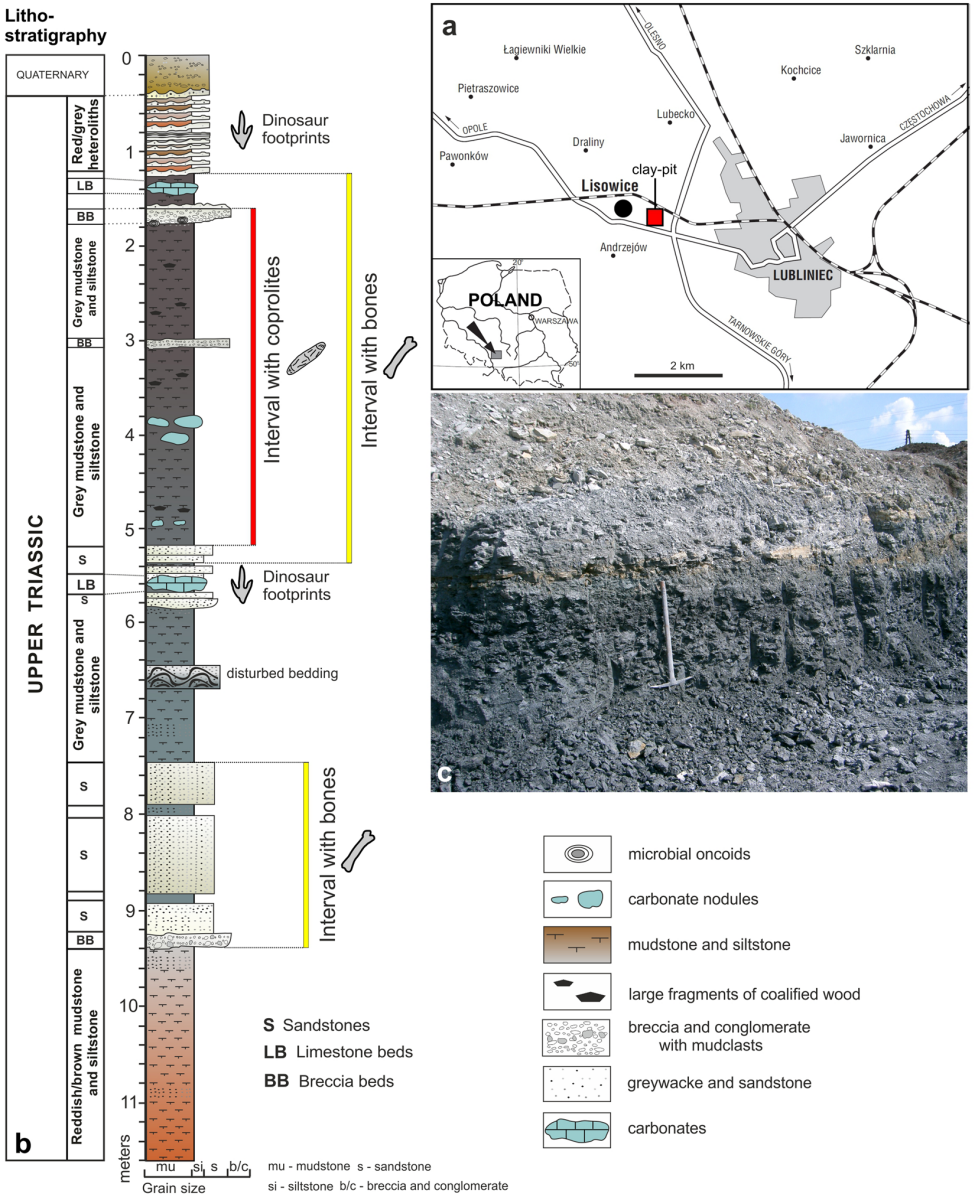
(**a**) Location of the study area within Poland and a roadmap of the Lubliniec area showing the location of Lisowice and the Lipie Śląskie clay-pit. (**b**) General lithostratigraphical column of the Upper Triassic (upper Norian-lowermost Rhaetian) succession from Lisowice (Lipie Śląskie clay-pit) with the position of the coprolite-bearing and bone-bearing beds. (**c**) Photograph of dark and organic-rich mudstones/siltstones from the upper part of the section from which the large coprolites were found.

**Figure 2:**
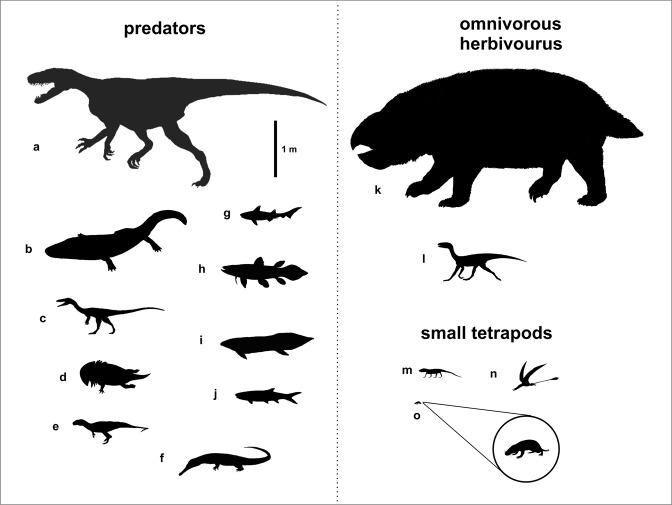
Sketch-drawing of the vertebrate faunal assemblage of the Lisowice site (modified from Niedźwiedzki)^10^. (**a**) Large, theropod-like predatory archosaur (*Smok wawelski*); (**b**) large temnospondyl amphibian (*Cyclotosaurus* sp.); (**c**) small predatory dinosaurs (Neotheropoda indet.); (**d**) temnospondyl amphibian (*Gerrothorax* sp.); (**e**) small basal crocodylomorph (Crocodylomorpha indet.); (**f**) small diapsid (Choristodere-like animal); (**g**) hybodont sharks (*Polyacrodus* and *Hybodus*); (**h**) coelacanth fish; (**i**) dipnoan fish (*Ptychoceratodus* sp.); (**j**) actinopterygian fish; (**k**) gigantic dicynodont; (**l**) dinosauriforms or early dinosaurs (Dinosauriformes indet. or Dinosauria indet.); (**m**) small lepidosauromorphs (Sphenodontia indet.); (**n**) pterosaurs (Pterosauria indet.); (**o**) early mammaliaform (*Hallautherium* sp.).

The material of *S. wawelski* is associated with numerous bones of a large dicynodont as well other vertebrates. Many of these bones show deep bite marks; one juvenile dicynodont fibula has had its distal head bitten off^12^. The size of the bite marks matches the teeth of *S. wawelski*^12^, which suggests that this predator was at least an occasional osteophage. We decided to investigate this possibility by analysing ten relatively large coprolites (87–250 mm long and around 30–50 mm wide; Table 1) from Lisowice by both classical analytic techniques and phase-contrast synchrotron microtomography (PPC-SRμCT). The latter has been shown to be an efficient method to visualize the full contents of coprolites in three dimensions^13^. In addition to *S. wawelski* and the aforementioned giant dicynodont, the fauna from Lisowice contains numerous small diapsids, archosauromorphs, temnospondyls, bony fishes and sharks^11,14,15^. Well-preserved vertebrate bones of these occur in two intervals (upper and lower), in total six horizons. The section with fossiliferous beds is about 12 meters thick and was exposed in the Lipie Śląskie clay-pit at Lisowice village near the town of Lubliniec in southern Poland^11,14,15^ (Fig. 1). The richest bone record is from the upper interval. The remains from this level are usually preserved in grey lenticular bodies of carbonate-rich siltstones and mudstones, which are most often covered with calcareous and pyritic crusts, or preserved within limestone concretions. More than 50 small to large coprolites were collected from this upper bone-bearing interval. The vertebrate fossil assemblage of the upper interval consists mainly of terrestrial rather than amphibious or aquatic tetrapods^10,11,16,17^ (Fig. 2). The most common bones are from a giant dicynodont, followed by archosaur and temnospondyl bones. The top predator in this assemblage is represented by theropod-like archosaur *S. wawelski*^11^ and the diversity of small and medium-sized reptilians is relatively high as indicated by the presence of: (1) pterosaurs –cranial elements, limb bones, vertebrae and teeth (Pterosauria indet.); (2) two species of dinosauriforms or early dinosaurs –cranial elements, vertebrae, limb and pelvic bones (Dinosauriformes indet. or Dinosauria indet.); (3) two or more species of small predatory dinosaurs –cranial elements, limb and pelvic bones (Neotheropoda indet.); (4) small crocodylomorph limb bones (Crocodylomorpha indet.); (5) choristodere-like vertebrae and limb bones (Diapsida indet.), skull bones, limb bones and teeth of lepidosauromorphs (Sphenodontia indet.) and numerous isolated bones and teeth of other, still unidentified, small diapsids (Diapsida indet.). Temnospondyls (*Cyclotosaurus* sp. and *Gerrothorax* sp.) are known from isolated skull bones, partial and complete jaws, numerous limb bones and bony dermal elements. The fish fauna identified based on macrofossils is rich and includes large coelacanths (skull elements, scales), medium to large dipnoan fish (mainly skull bones and tooth plates of *Ptychoceratodus* sp.) and medium-sized actinopterygian fish (skull bones, teeth, numerous scales). The record of vertebrate microfossils and small fossils is dominated by remains of aquatic vertebrates and are comprised primarily of scales (or other dermal elements) and teeth of Actinopterygii, along with teeth and dermal denticles of the shark genera *Polyacrodus* and *Hybodus*^18–20^, but also teeth of archosaurs and postcranial fossils of early anurans^21^.

**Table 1:** List of analysed coprolites including specimen numbers, sizes, inclusions and analytic methods.

Specimen	Size (in mm)	Inclusions and other elements	Analytic methods	Comments
*ZPAL V.33/341*	Length: 87 Width: 31	A serrated tooth of *S.wawelski*, abundant bone fragments including temnospondyl bone, pyritzed microbial colonies, gas bubbles, compound of fibers	PPC-SRμCT	
*ZPAL V.33/344*	Length: 92 Width: 33	A serrated tooth of *S.wawelski*, a small serrated tooth, abundant bone fragments. Pyritized microbial colonies, gas bubbles	PPC-SRμCT	
*ZPAL V.33/345*	Length: 91 Width: 29	Abundant bone fragments including ribs of unknown prey, (?) juvenile dicynodont. Pyritized microbial colonies, gas bubbles	PPC-SRμCT	
*ZPAL V.33/340*	Length: 125	Bone fragments, microbial structures	SEM, thin sections, polish surfaces, dissolved	
*ZPAL V.33/342*	Length: 94	Bone fragments, microbial structures	SEM, dissolved	Incomplete
*ZPAL V.33/343*	Length: 130	Bone fragments	surface observations, polished surface	Incomplete
*ZPAL V.33/346*	Length: 116	Bone fragments (very dicynodont-like bones), microbial tunnels, microbial attack on the surface	SEM, thin sections, polished surface, dissolved	
*ZPAL V.33/600*	Length: 118	A tooth of *S. wawelski* without enamel, fish remains, bone fragments, microbial structure	SEM, thin sections, polished surface, dissolved	
*ZPAL V.33/604*	Length: 176	Bone fragments, microbial structures	SEM, observation of broken fragments, polished surface	
*ZPAL V.33/1890*	Length: 250	Bone fragments	surface observations	

*Width refers to the maximum width.

A recent U-Pb dating of a single zircon grain (Ion Microprobe SHRIMP IIe/MC), recovered from the sandstone bed positioned below the upper fossil-bearing interval, yielded an absolute age of 211 ± 3 Ma^17^. This means that the upper bone-bearing interval must be younger than the zircon grain, which provides a maximum deposition age of the layers in which it was found. The boundary between the Norian and Rhaetian stages is currently defined at the age of ~208.5 million years (see^22^); the zircon date thus suggests a late Norian to earliest Rhaetian age for the Lisowice fauna. This is in agreement with the faunal evidence. The presence of diversified dinosaur fauna and double-rooted, early mammaliaform teeth (*Hallautherium* sp.), point to a late Norian-earliest Rhaetian age of this assemblage^23,24^. Additional data from U-Pb dating of detrital zircons will be published soon.

A few trackways and numerous isolated tetrapod tracks have been found in sandstone intercalations located at the bottom and near the top of the section (Fig. 1b). The ichnoassemblage is dominated by tridactyl footprints of different sizes. The most common are *Grallator*-like and *Anchisauripus*-like footprints left by two or more dinosaur taxa. Large, about 40–50 cm long, tridactyl footprints, which morphologically resemble the ichnogenus *Eubrontes* were also found at Lisowice^25^.

## Results

**Figure 3:**
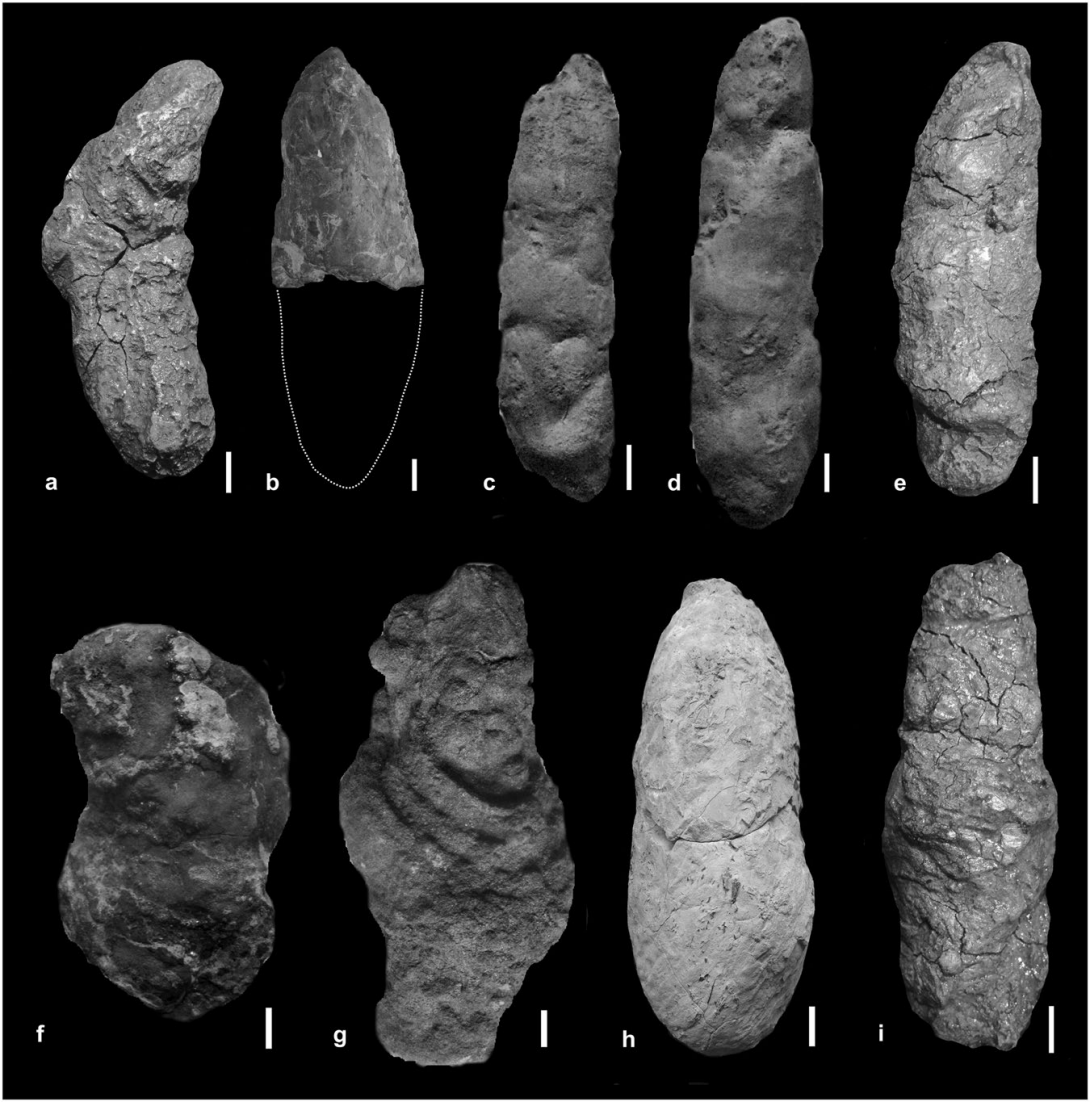
Large to medium-sized, elongated, bone-bearing and phosphate-rich *S. wawelski* coprolites from Lisowice, Upper Triassic, Poland. (**a**) ZPAL V.33/344. (**b**) ZPAL V.33/342. (**c**) ZPAL V.33/346. (**d**) ZPAL V.33/604. (**e**) ZPAL V.33/345. (**f**) ZPAL V.33/600. (**g**) ZPAL V.33/343. (**h**) ZPAL V.33/340. (**i**) ZPAL V.33/341. (**a**–**e,h,i**) Elongated specimens. (**f,g**) Elongated but slightly more irregular specimens. Scale bars: 1 cm.

**Figure 4:**
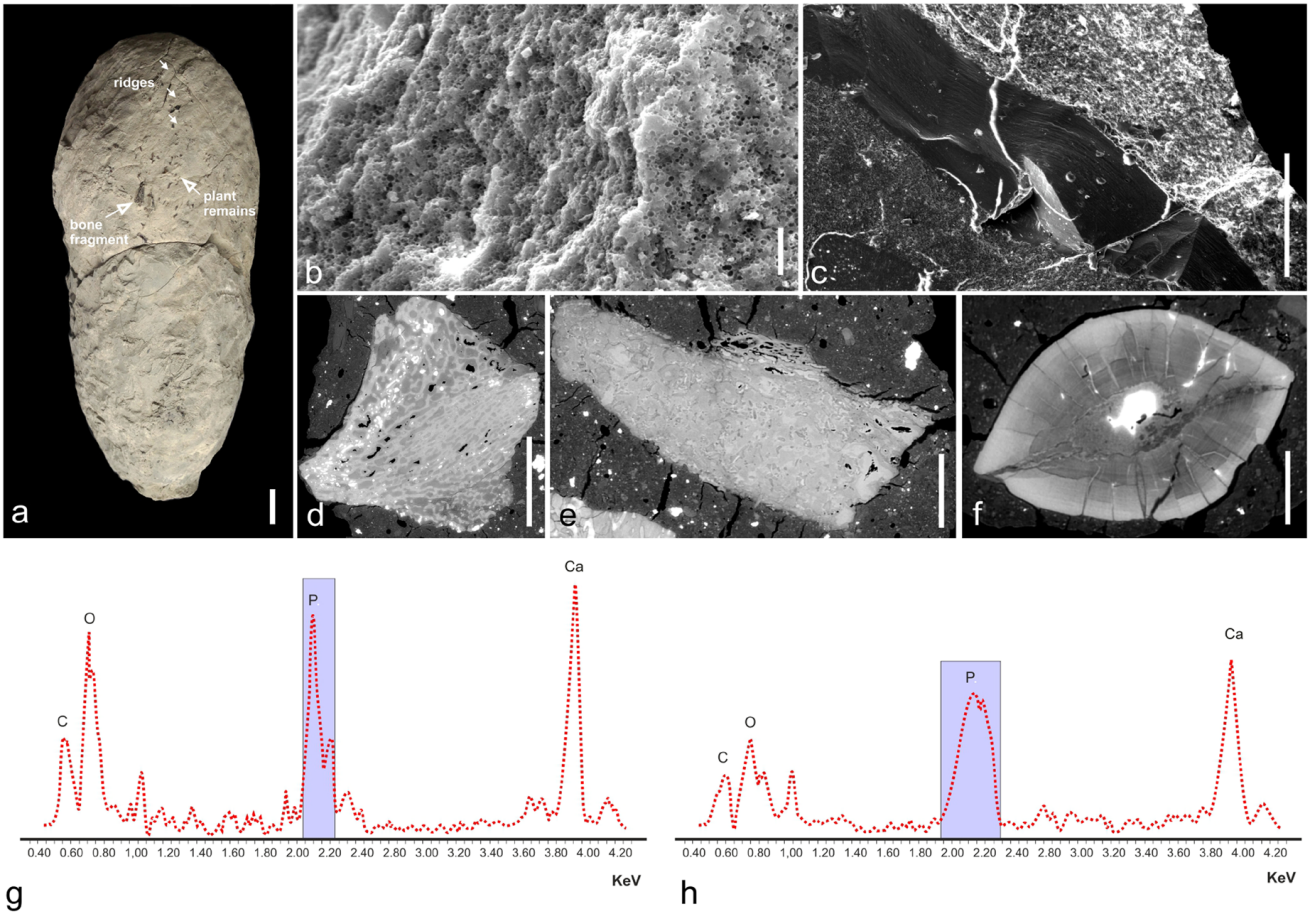
Inclusions and matrix composition of the large coprolites. (**a**) Specimen ZPAL V.33/340 with bone and plant fragments exposed on the surface. (**b,c**) SEM images of coprolite matrix with micron-sized spherical structures (**b**) and section of a fish scale (**c**) preserved in the coprolite matrix. (**d**–**f**) Virtual sections showing bone inclusions (**d**,**e** - fragments of bones; **f** - tooth). (**g,h**) EDS spectra of matrix from two coprolites displaying a calcium phosphatic composition (g – ZPAL V.33/600; h – ZPAL V.33/604).  Scale bars: a - 10 mm; b - 0.2 mm; c - 1 mm; d - 10 mm; e - 3 mm; f - 2 mm.

The coprolite material described here (Fig. 3) was collected from the upper bone-bearing interval (Fig. 1) with carbonaceous greenish and grey fluvial claystone, siltstones and mudstones, interbedded with cross- or horizontally-stratified greywacke sandstones and local conglomerates^16^. All studied coprolites are elongated, oval in cross section, and typically have a blunt and a tapered end (Fig. 3, Table 1). The different morphology of the ends, together with structures of compression, provide evidence for the direction of movement through the gut, with the blunt end being excreted first^26^. The coprolites are composed of a well-mineralized matrix with abundant micron-sized bacterial pseudomorphs (round spheres and pits), visible in SEM (Fig. 4b). As evidenced by EDS spectra (Fig. 4g,h), the matrix contains elevated concentrations of phosphorus, calcium and sodium, and lower concentrations of silicon and aluminium, relative to the host sediment. A large fraction of the phosphate probably derived from dissolved bone apatite and soft tissues of prey. This excess of phosphate likely favoured an early bacterial-induced mineralisation, which is thought to play a major role in the preservation of the faeces and the food inclusions they contain^3,27–29^.

**Figure 5:**
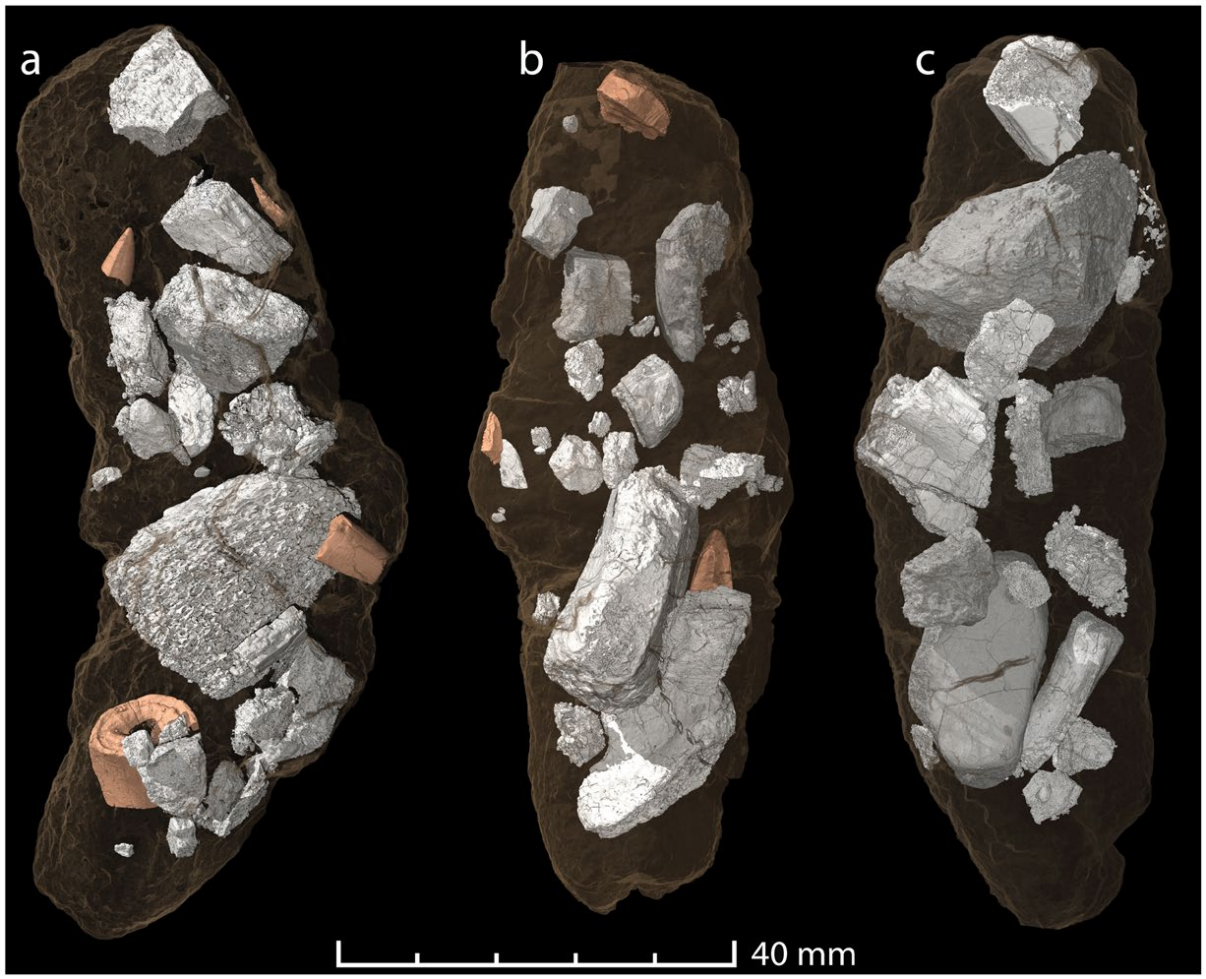
Virtual reconstructions of the three scanned specimens (semi-transparent), showing the enclosed bones (white) and tooth inclusions (orange). Gross morphology and contents of coprolites ZPAL V.33/344 (**a**); ZPAL V.33/341 (**b**), and ZPAL V.33/345 (**c**).

The three synchrotron-scanned coprolites contain conspicuous amounts of bone fragments (Fig. 4) that make up approximately half of the total coprolite volume (Fig. 5), something that is previously unrecorded from the otherwise relatively rich coprolite record of the Triassic^30,31^. A few of the bones exhibit grooves and pits that are similar to bite traces found on dicynodont bones from the locality (Fig. 5)^10,12^. The bones are fragmented and range from submillimetre to centimetre scale. This size variation is linked to the original dimensions of the bones, their inherent resistance to damage (e.g. compact bone versus spongy bone) and probably to processing through the digestive tract (e.g. size after biting, subsequent processing, and time spent in the digestive tract). The cortical bone tissues are variously vascularized, variably remodelled and some contain lines of arrested growth that are either evenly spaced or with shortening gaps toward the exterior of the bone (see Supp. Fig. 1). These histological features alone do not allow an attribution of the bones to prey species. However, they do indicate that juveniles, adults, fast- and slow growing animals of both terrestrial and aquatic taxa were all present amongst the prey.

**Figure 6:**
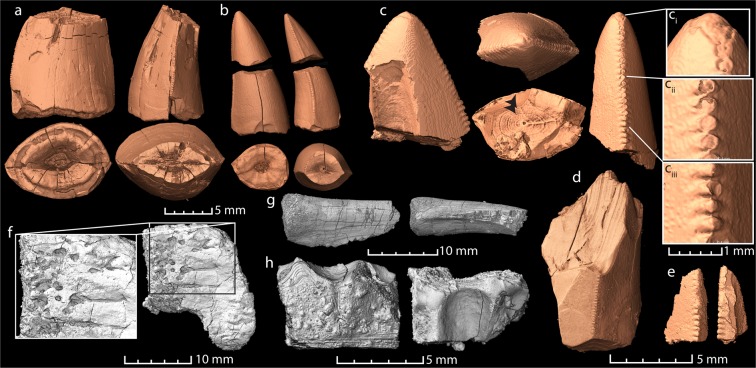
Virtual reconstructions of the serrated tooth remains and a selection of bones found in the scanned coprolites. (**a**) Images of a piece of a large, well-worn tooth in different views. (**b**) Two pieces of a small serrated tooth in different views (found separately, but belonging to the same tooth, in the same coprolite). Note the pulp cavity (visible in cross sections) that is thinning out toward the tip. (**c**) Tip from a broken tooth with serration and visible incremental growth lines (black arrows). (c_i_–c_iii_) Enlargement of the tip and serrations of the tooth tip. Note the wear of the tip (c_i_) and the topmost serrations (c_ii_) in contrast to the more complete basal serrations (c_iii_). (**d**) Fragment of a basal part of a tooth crown, likely from the same tooth as (**c**,**e**). (**e**) Small tooth splinter with serrations. (**f**) Flat bone fragment with bite marks (enlarged). (**g**) Incomplete rib with longitudinal ridge, possibly from a small archosaur. (**h**) Dermal bone fragment with ornamentation derived from a temnospodyl amphibian. The images are from the coprolite specimens ZPAL V.33/344 (**a**–**b**); 2PAL v33 341 (**c**–**f**,**h**); and ZPAL V.33/345 (**g**).

Recognizable bones include an ornamented dermal bone of a temnospondyl amphibian (Fig. 6h) and a piece of a limb bone that tentatively belonged to an early juvenile dicynodont (? humerus). Several fragments of ribs, of which one displays a longitudinal ridge suggesting it may derive from an archosaur prey item (Fig. 6g), are present in coprolite ZPAL V.33/344.

In coprolite ZPAL V.33/344, one piece of a serrated large tooth crown and two pieces of a smaller, more complete tooth are found. The larger specimen lacks the apical tip and displays wear facets on the serrations, cracks and grooves in the dentine, and generally uneven surfaces (Fig. 6a). In contrast, the smaller specimen shows little sign of wear except for the surfaces of abrupt breakage (Fig. 6b). A tooth tip, a large fragment of the basal part of the tip, and a small splinter with serrations of presumably the same tooth were found in coprolite ZPAL V.33/341 (Fig. 6c). These fragments show similar preservation to the small and well-preserved tooth in ZPAL V.33/344, although the tip and serrations display wear facets. All three teeth are serrated, rounded towards the base, and flattened toward the tip. A fourth serrated tooth was found in coprolite ZPAL V.33/600, further indicating that the presence of teeth is not rare in the coprolites. This tooth is much etched from digestive acids with the enamel being completely removed (cf. teeth in scat of modern crocodiles)^32^. The tooth from coprolite ZPAL V.33/341, the bigger tooth in coprolite ZPAL V.33/341, and the etched tooth from ZPAL V.33/600 all match the size, morphology and wear of teeth of subadult *S. wawelski* individuals. The smaller tooth, however, is too small to have derived from a large subadult individual of *S. wawelski* and is similar to teeth of a small archosaur found in the deposits.

Other contents from the coprolites include: fish remains (in coprolite ZPAL V.33/600), burrowing traces of annelids, gas escape voids, enigmatic inclusions, detrital grains, a possible charcoal fragment, structures composed of parallel-running fibres of animal or plant origin, a tube-shaped inclusion of unknown origin, and a small hooklet of, perhaps, plant or arthropod origin (Supp. Fig. 2). The detrital mineral grains are dominated by quartz, but include other minerals as well (e.g. plagioclase, biotite). These grains are angular to subrounded with a size range of about 0.05 to 0.3 mm (silt to fine to medium-grained sand) and were most likely accidently ingested.

## Discussion

The similarities in shape, the large sizes, and the contents of the coprolites suggest that they were produced by one and the same species that occupied a position as apex predator in the ecosystem. *S. wawelski* is the only taxon from the locality that matches these criteria. The three teeth of *S. wawelski* were probably involuntarily ingested as the coprolite-producing individual broke its own teeth during feeding (although cannibalism cannot be completely ruled out). Most wear surfaces of the two large teeth from the scanned coprolites are from *in vivo* wear in the mouth, and there is little sign of etching by digestive acids. The high *in vivo* abrasion seen on these teeth and on individual teeth in Lisowice suggests that *S. wawelski* often used its teeth on hard elements, such as bones. An osteophagous behaviour is also supported by bone-rich regurgitalites from the same bone-bearing beds^10^ (Supp. Fig. 3). The regurgitalites are composed of accumulated and angular bone fragments that are larger than the bone pieces in the coprolites. This suggests that *S. wawelski* regurgitated larger, indigestible, fragments in a manner comparable to modern birds such as owls.

There is a marked difference in degradation between the enamel-stripped tooth from ZPAL V.33/600 and the other more well-preserved teeth. Also some bones are much degraded and hardly recognizable, whereas others are very well preserved. This suggests that the food residues had different duration times in the digestive tract and that they were mixed over time. Variations in food retention time occur interspecifically (cf. the slow digestion of crocodilians versus the fast digestion of mammals) but can also differ in the same species (or individual) depending on environmental conditions, food type and availability^33–37^.

**Figure 7:**
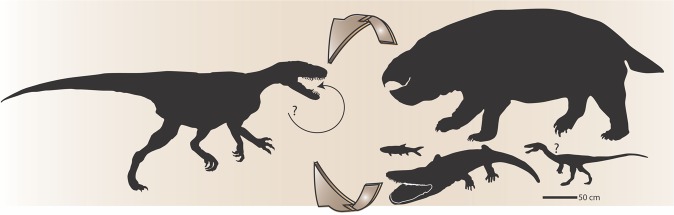
*Smok wawelski* together with prey animals inferred from coprolite contents and bones with bite marks. Arrows indicate predator-prey relations, based on: a small tooth of Theropoda indet. in coprolite ZPAL V.33/344; tooth marks on dicynodont bones and putative dicynodont bones in coprolites; teeth of *S. wawelski* in several coprolites; temnospondyl dermal bone in coprolite ZPAL V.33/341; fish remains in ZPAL V.33/600.

Recent archosaurs, i.e. crocodilians and birds, typically ingest prey with little mastication and can fully digest bones^32,38^. Gut contents associated with theropod skeletons often contain whole bones or partial skeletons (e.g.^39–41^). This suggests that many theropods ingested prey items with a minimum of oral processing. As evidenced by coprolites, regurgitalites and bite marks on dicynodont bones, *S. wawelski* appears to have taken a different approach to osteophagy, fragmenting the bones by repeated biting in a manner somewhat reminiscent of a hyena. The only theropod dinosaurs known to show evidence of similar feeding adaptations are the large-bodied tyrannosaurids of the Late Cretaceous^2,42^. Tyrannosaurs like *Tyrannosaurus rex* were able to bite deep into bones due to high bite forces and tooth pressures, tooth morphology, and repeated, localized biting^42^. It has also been argued that the distinct fragmentation and angularity of bones within a coprolite probably produced by *T. rex* reflect extensive bite- rather than gizzard-induced breakage^2,3^. The coprolites of *S. wawelski* contain at least as much bone per volume as that of *T. rex*, and the size fractions of bones and the degree of etching are very similar. Even though *S. wawelski* is considerably smaller than these tyrannosaurs, we conclude that it occupied a similar ecological role of osteophagous top predator (Fig. 7). Like many convergences in the body fossil record, this extends the record of bone-chewing osteophagy among archosaurs by 140 million years. Since coprolites contain direct evidence on feeding that can enable reconstructions of ancient trophic relations^2,15,26,43–45^, we suggest that they represent one of the best targets to investigate a potentially underestimated occurrence of bone chewing among Mesozoic dinosaurs and archosaurs.

## Methods

### Phase-contrast synchrotron microtomography

Three coprolites were scanned using propagation phase-contrast synchrotron microtomography (PPC-SRμCT) at beamline ID19 of the European Synchrotron Radiation Facility (ESRF) in Grenoble, France. The coprolites were scanned in vertical series of 4 mm, in so-called half acquisition mode meaning that the center at rotation was set at the side of the camera field of view, resulting in a doubling of the reconstructed field of view. The propagation distance, or the distance between the sample on the rotation stage and the camera, was set at 2800 mm. The camera was a sCMOS PCO edge 5.5 detector, mounted on an optical device bringing an isotropic voxel size of 13.4 μm, and coupled to a 1000-μm thick GGG:Eu (Gadolinium gallium garnet doped with europium) scintillator. The beam produced by a W150 wiggler (11 dipoles, 150 mm period) with a gap of 50 mm was filtered with 2.8 mm aluminum and 6 mm copper. The resulting detected spectrum had an average energy of 113 keV. Each sub scan was performed using 6000 projections of 0.02 s each over 360 degrees.

The reconstructions of the scanned data were based on a phase retrieval approach^46,47^. Ring artefacts were corrected using an in-house correction tool^48^. Binned versions (bin2) were calculated to allow faster processing and screening of the samples since the full resolution data was large. The final volumes consist in stacks JPEG2000 images that were subsequently imported and segmented in the software VGStudio MAX version 3.0 (Volume Graphics Inc.).

### Optical microstructure observations

Five coprolites were studied in detail based on thin sections. Standard petrographic thin sections were prepared and later examined under an optical microscope (NIKON Eclipse LV100 POL). Images were collected using a NIKON digital camera.

### Scanning electron microscopy coupled with energy-dispersive X-ray spectroscopy

Material from five coprolites was analysed in a Phillips XL-20 scanning electron microscope equipped with the EDS detector ECON 6, system EDX-DX4i and a backscatter electron (BSE) detector for Compo or Topo detection (FEI product). This instrument was operated at an accelerated voltage of 25 kV, a beam current of 98–103 nA, and a spot diameter of 4 μm. SEM images were collected.

## Supplementary information

supp info
