# Supplementary material for: Tyrannosaurid-like osteophagy by a Triassic archosaur
Source: Sci Rep. 2019 Jan 30;9:925. doi: 10.1038/s41598-018-37540-4 (PMC6353991; doi:10.1038/s41598-018-37540-4)
Supplement: Supplementary file 1 — supp info [file 41598_2018_37540_MOESM1_ESM.docx]

**SUPPLEMENTARY INFORMATION for:**

**Tyrannosaurid-like osteophagy by a Triassic archosaur**

Martin Qvarnström^1^, Per E. Ahlberg^1^ and Grzegorz Niedźwiedzki^1^*

^1^Department of Organismal Biology, Evolutionary Biology Centre, Uppsala University, Norbyvägen 18A, 752 36 Uppsala, Sweden

*Corresponding author

E-mail address: grzegorz.niedzwiedzki@ebc.uu.se (G. Niedźwiedzki)

**Appendix S1. Details about the locality and fossil horizon.**

The most productive fossiliferous beds were well exposed between 2007-2014 (but are now under water) in the Lipie Śląskie clay-pit at Lisowice^10,11,16^. According to comparisons with lithologic descriptions of boreholes^49,50^ this unit is characteristic for the upper Zbąszynek and lower Wielichowo Beds from the north-western and central parts of Poland^10,11,16,51-54^. The Upper Triassic deposits from Lisowice are also, in part, similar to a local lithostratigraphic unit named the Lisów Beds (or Lisów Formation) known from the two nearly located Solarnia IG-1 and Lubliniec IG-1^55^ boreholes in Silesia, and also to the so-called Woźniki Formation exposed in some places near Lubliniec^55^. This simple lithostratigraphic correlation is also consistent with biostratigraphic data (conchostracans and palynomorphs) collected from the Lisowice exposure and their correlation with borehole records from the Polish Lowland (see below).

Identifications of palynomorph fossils in the grey and organic-rich strata at the Lipie Śląskie clay-pit^10,11,16^ suggest a correlation of this unit with the *Corollina meyeriana* Zone, Subzone IVb (upper part) and Subzone IVc (middle-upper Norian and uppermost Norian-lowermost Rhaetian respectively^56-58^) and also *Ricciisporites tuberculatus* Zone (middle-upper Rhaetian in age^56-58^), which are well-defined based on core material from the central and northern part of Poland^53,54,56,58^. The upper Zbąszynek Beds apparently continues into the upper Arnstad, upper Löwenstein, Trossingen, and lower part of the Exter Formation of the upper Middle to Upper Keuper (upper Norian–Rhaetian) in the middle-eastern part of the Germanic Basin^53^, which contains the geologically oldest fossils of early mammaliaforms and a diversified fauna of early dinosaurs^59-61^. In the Polish geological literature, the lithostratigraphic unit represented at the Lipie Śląskie clay-pit has been referred to as Norian^62^ or Rhaetian “*sensu polonico*” ^51-53^. Recently, some authors^63,64^ included the bone-bearing deposits from Lisowice to a re-defined Grabowa Formation and suggest middle Norian age of this tetrapod fauna. This is mainly based on chemostratigraphical correlations with support of palynological studies (e.g. identification of *Corollina meyeriana* Subzone IVb in Lipie Śląskie clay-pit section). Such an interpretation is contradicted by paleontological and biostratigraphical data collected from Lisowice section (see below) and other bone-bearing Late Triassic sites in Silesia.

The bone and coprolite-bearing intervals are rich in organic remains and macrofossils of plants. The dominant plant species at Lisowice is a conifer closely similar to *Brachyphyllum, Pagiophyllum* or *Hirmeriella*, as is the case with the upper Norian-Rhaetian and Lower Jurassic floras of the region^65,66^. The second most common plant species is represented by twigs and seeds closely similar to Stachyotaxus septentrionalis **(Agardh, 1823), a taxon characteristic** for the Rhaetian of Greenland and Scania^67-69^. Other plant fossils, which are currently examined in detail, are represented by cycadophytes, ginkgophytes, and pteridosperms^70^.

Several other findings are also suggestive of an early Rhaetian age including: numerous cuticle fragments and rare leaf fragments or fructifications (*Peltaspermum rotula* Harris, 1937) of the typical Rhaetian seed-fern *Lepidopteris* *ottonis* (Goeppert, 1836) ^71-75^; the isoëtalean macrospores *Trileites* cf. *pinguis* (Harris, 1935) and *Horstisporites bertelseni* Fuglewicz, 1977^76,77^; and the conchostracans *Gregoriusella polonica* Kozur, Niedźwiedzki *et* Sulej, 2010, *Euestheria* sp., and *Shipingia* sp. collected mainly from layers located below upper bone-bearing interval^16,78^. Monospecific assemblages with *G*. *polonica* are known from the lower Exter Formation of northern Germany (early Rhaetian) and the upper Redonda Formation of New Mexico (latest Norian-early Rhaetian), with this species occurring above the *Shipingia olseni* Zone, which is correlated with the late Norian Sevatian substage^64,79^. The *S. olseni* Zone is followed by a short interval that contains both abundant specimens of the very small form *G. polonica* and the last specimens of *S. gerbachmanni*; this interval could be either latest Norian or earliest Rhaetian^80^.

**Appendix S2.** **Supporting References.**

49. Dadlez, R., Kopik, J. Problem retyku w zachodniej Polsce na tle profile w Książu Wielkopolskim. *Kwartalnik Geologiczny* **7**, 131–158 (1963).

50. Deczkowski, Z. Noryk–Retyk, Jura dolna. *In:* S. Marek and M. Pajchlowa (eds), Epikontynentalny perm i mezozoik w Polsce. *Prace Państwowego Instytutu Geologicznego* **153**, 174–235 (1997).

51. Franz, M., Bachmann, G.H., Beutler, G. Retyk *sensu polonico versus* Rhaet *sensu germanico* – new results. *Schriftenreihe der Deutschen Gesellschaft für Geowissenschaften* **53**, 99–100 (2007a).

52. Franz, M., Bachmann, G. H., Beutler, G. Sedimentology and Facies of the Polish Retyk and the German Arnstadt and Exter Formations (Norian, Rhaetian) in the eastern Central European Basin (CEB). *Schriftenreihe der Deutschen Gesellschaft für Geowissenschaften* **53**, 101 (2007b).

53. Franz, M. *Litho- und Leitflächenstratigraphie, Chronostratigraphie, Zyklo- und Sequenzstratigraphie des Keupers im östlichen Zentraleuropäischen Becken (Deutschland, Polen) und Dänischen Becken (Dänemark, Schweden)*. Dissertation zur Erlangung des akademischen Grades doctor rerum naturalium (Dr. rer. nat.) vorgelegt der Naturwissenschaftlichen Fakultät III der Martin-Luther-Universität Halle-Wittenberg, verteidigt am 08.12.2008: 1–198 (2008).

54. Pieńkowski, G., Niedźwiedzki, G., Waksmundzka, M. Sedimentological, palynological, and geochemical studies of the terrestrial Triassic–Jurassic boundary in north-western Poland. *Geological Magazine* **149**, 308–332 (2012).

55. Haisig, J., Kotlicki, S., Wilanowski, S., Żurek, W. *Objaśnienia do szczegółowej mapy geologicznej Polski, Arkusz Lubliniec.* 46 pp. Wydawnictwa Geologiczne, Warszawa (1983).

56. Orłowska-Zwolińska, T. Palinostratygrafia epikontynentalnych osadów wyższego triasu w Polsce. *Prace Instytutu Geologicznego* **104**, 1–88 (1983).

57. Orłowska-Zwolińska, T. Palynological zones of the Polish epicontinental Triassic. *Bulletin of Polish Academy of Sciences, Earth Sciences* **33**, 107–119 (1985).

58. Fijałkowska-Mader, A. A record of climatic changes in the Triassic palynological spectra from Poland. *Geological Quarterly* **59,** 615–653 (2015).

59. Clemens, W.A. Rhaeto-Liassic mammals from Switzerland and West Germany. *Zitteliana, Abhandlungen der Bayerischen Staatssammlung für Paläontologie und Historische Geologie* **5**, 51–92 (1980).

60. Kielan-Jaworowska, Z., Cifelli, R.L., Luo, Z.-X. 2004. *Mammals from the Age of Dinosaurs: Origins, Evolution, and Structure*. 630 pp. Columbia University Press, New York.

61. Seegis, D. Tetrapoden. *In*: Deutsche Stratigraphische Kommission (ed.), Stratigraphie von Deutschland, Keuper-Band. *Courier Forschungsinstitut Senckenberg* **253**, 50–51 (2005).

62. Szulc, J., Gradziński, M., Lewandowska, A., Heunisch, C. The Upper Triassic crenogenic limestones in Upper Silesia (southern Poland) and their paleoenvironmental context. *In*: A.M., Alonso-Zarza, L.H., Tanner (eds.), Paleoenvironmental Record and Applications of Calcretes and Palustrine Carbonates. *Geological Society of America Special Papers* **416**, 133–151 (2006).

63. Szulc, J., Racki, G. Grabowa Formation – the basic lithostratigraphic unit of the Upper Silesian Keuper. *Przegląd Geologiczny* **63**, 103–113 (2015).

64. Lucas, S.G. Age and correlation of Late Triassic tetrapods from southern Poland. *Annales Societatis Geologorum Poloniae* **85**, 627–635 (2015).

65. Clement–Westerhof, J.A., Van Konijnenburg–Van Cittert, J.H.A. New data on the fertile organs leading to a revised concept of the Cheirolepidiaceae. *Review of Palaeobotany and Palynology* **68**, 147–179 (1991).

66. Reymanówna, M. Two conifers from the Liassic flora of Odrowaz in Poland. *In*: J. Kovar–Eder (ed.), *Palaeovegetational Development in Europe and Regions Relevant to its Palaeofloristic Evolution, Proceedings of the Pan-European Palaeobotanical Conference, Vienna, 19-23 September 1991*, 307–311(1992).

67. Harris, T.M. 1932. The fossil flora of Scoresby Sound East Greenland. Part 4: Gingkoales, Coniferales, Lycopodiales and isolated fructifications. *Meddelelser om Grønland* **112**, 1–12.

68. Arndt, S. Morphologie und Systematik ausgewählter Mesozoischer Koniferen. *Palaeontographica B* **262**, 1–23 (2002).

69. Taylor, T.N., Taylor, E.L., Krings, M. *Paleobotany – the biology and evolution of fossil plants*. Academic Press, Burlington ⁄ Elsevier, Amsterdam, XXII + 1230 pp. (2009).

70. Wawrzyniak, Z. *Późnotriasowa flora z Lipia Śląskiego*, 76 pp. MSc Thesis, Wydział Nauk o Ziemi, Uniwersytet Śląski, Sosnowiec (2010).

71. Staneczko, K. Nowe dane paleobotaniczne na temat górnego triasu z Lipia Śląskiego koło Lublińca (południowa Polska). *Geo-Sympozjum Młodych Badaczy Silesia 2007*, 155–168 (2007).

72. Wawrzyniak, Z., Ziaja, J. 2009. Wstepne wyniki badan gornotriasowej makroflory Lipia Śląskiego, Polska. *Geologia* **35**, 105–106.

73. Wawrzyniak, Z. 2011. The Upper Triassic cuticles from Lipie Śląskie (South Poland) *In:* M. Bąk, M.A. Kaminski, A. Waśkowska (eds), Integrating Microfossil Records from the Oceans and Epicontinental Seas. *Grzybowski Foundation Special Publication* **17**, 140–141.

74. Pacyna, G. Plant remains from the Polish Triassic. Present knowledge and future prospects. *Acta Palaeobotanica* **51**, 3–33 (2014).

75. Zatoń, M. *et al.* Coprolites of Late Triassic carnivorous vertebrates from Poland: An integrative approach. *Palaeogeography, Palaeoclimatology, Palaeoecology* **430**, 21–46 (2015).

76. Fuglewicz, R. New species of megaspores from the Trias of Poland. *Acta Palaeontologica Polonica* **22**, 405–431 (1977).

77. Fuglewicz, R., Śnieżek, P. Upper Triassic megaspores from Lipie Śląskie near Lubliniec. *Przegląd Geologiczny* **28**, 459–461 (1980).

78. Kozur, H.W., Weems, R.E. The biostratigraphic importance of conchostracans in the continental Triassic of the northern hemisphere. *In:* S.G. Lucas (ed.) The Triassic Timescale. *Geological Society of London Special Publications* **334**, 315–417 (2010).

79. Lucas, S.G., Tanner, L.H., Kozur, H.W., Weems, R.E., Heckert A.B. The Late Triassic timescale: Age and correlation of the Carnian–Norian boundary. *Earth-Science Reviews* **114**, 1–18 (2012).

80. Barth, G.K., Kozur, H. A latest Norian age for insect-bearing beds of the Fuchsberg andLangenberg near Seinstedt, northern foreland of the Harz Mountains (Lower Saxony, Germany). *Fossil Record 3. New Mexico Museum of Natural History and Science, Bulletin* **53**, 157–165 (2011).

**Appendix S3.** **Supporting Figures.**


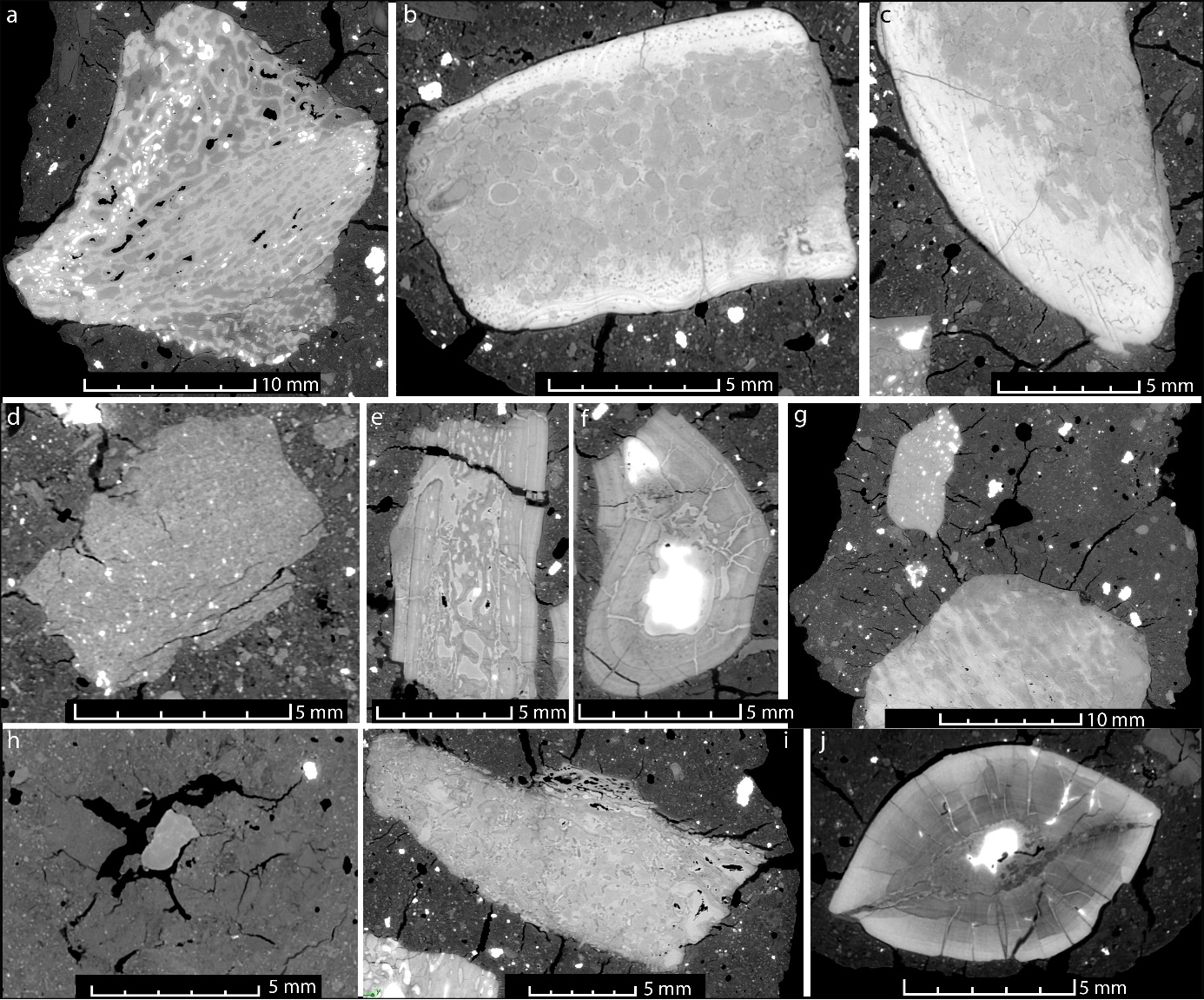


**Supp. Fig. 1.** **Virtual sections showing bone inclusions from the scanned *S. wawelski* coprolites. a.** A large bone fragment with complex morphology and thin cortical bone that likely derives from a juvenile dicynodont (ZPAL V.33/345). **b-c.** A large, heavily remodelled, bone fragment displaying osteocytes and vascularization. The wavy structure of the cortical bone and the high density suggest that it derives from a temnospondyl prey (ZPAL V.33/345). **d.** Bone heavily degraded from digestive etching. Note that the internal morphology (and histology) is almost unrecognizable (ZPAL V.33/345). **e.** Cross section of rib? (ZPAL V.33/345). **f.** Cross section of rib? (ZPAL V.33/345).  **g.** Bone inclusions and cracks developed in the coprolite matrix around the bones indicating desiccation (and shrinking) of the faeces before burial (ZPAL V.33/341). **h.** Bone and casts of completely dissolved bone (ZPAL V.33/344). **i.** Remodelled bone with highly vascularized cortex (ZPAL V.33/344). **j.** Cross section through the serrated tooth with plenty of cracks developed in the dentine (ZPAL V.33/344).


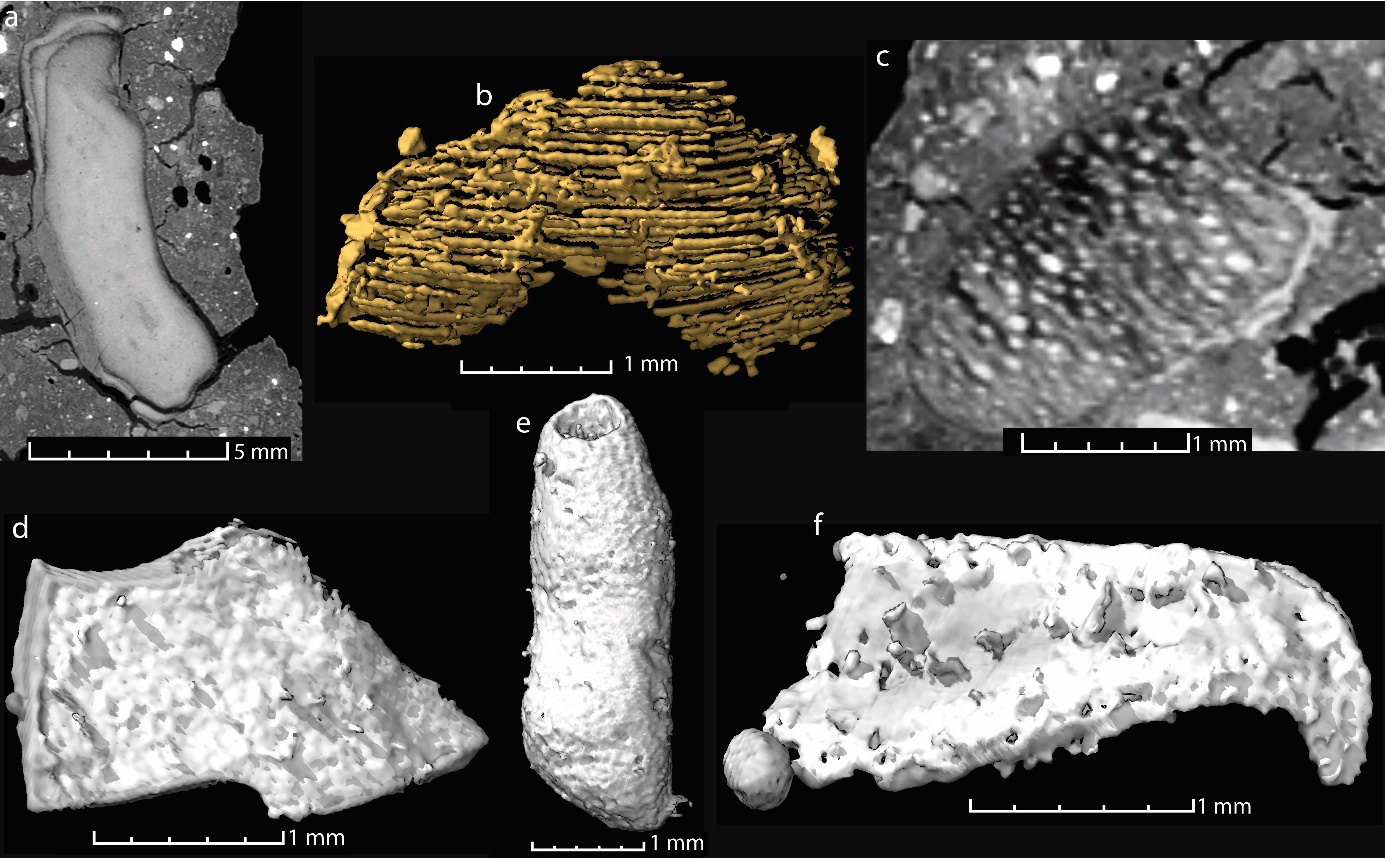


**Supp. Fig. 2.** **Other inclusions from *S. wawelski* coprolites. a.** A possible charcoal fragment (ZPAL V.33/345). **b-c.** A structure composed of parallel-running, 40-80 µm thick fibres, of animal or plant origin (ZPAL V.33/344). **d.** A low-density structure of unknown origin (ZPAL V.33/345). **e.** Tube-shaped inclusion of unknown origin (ZPAL v33 345). **f.** Small hooklet of, perhaps, plant or arthropod origin (ZPAL V.33/345).


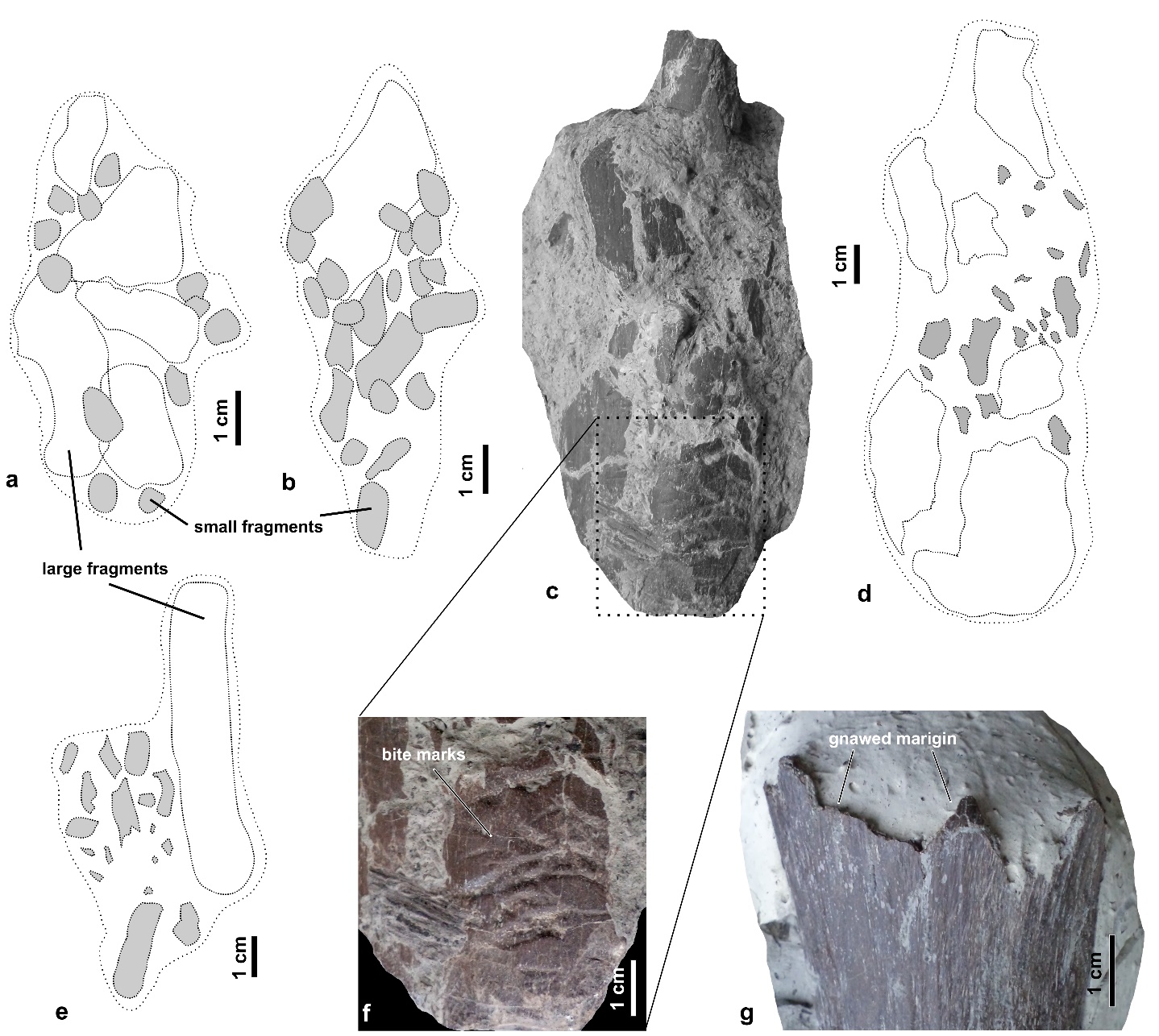


**Supp. Fig. 3.** **Supposed regurgitalites of *S. wawelski*. a, b, e.** Schematic drawings (general shape, composition) of regurgitalites with bone fragments. **c, d.** A large regurgitalite specimen (ZPAL V.33/704) containing bone fragments with sharp margins. **f.** Enlargement of bone fragment with traces of tooth marks. **g.** Dicynodont bone fragment with a sharp, probably gnawed, margin.
